# Chemokine-Binding Proteins Encoded by Parapoxvirus of Red Deer of New Zealand Display Evidence of Gene Duplication and Divergence of Ligand Specificity

**DOI:** 10.3389/fmicb.2019.01421

**Published:** 2019-06-25

**Authors:** Saeed Sharif, Norihito Ueda, Yoshio Nakatani, Lyn M. Wise, Sheree Clifton, Zabeen Lateef, Andrew A. Mercer, Stephen B. Fleming

**Affiliations:** Virus Research Unit, Department of Microbiology and Immunology, University of Otago, Dunedin, New Zealand

**Keywords:** chemokine, chemokine-binding protein, parapoxvirus, parapoxvirus of red deer in New Zealand, cell migration

## Abstract

Parapoxvirus of red deer in New Zealand (PVNZ) is a species of the *Parapoxvirus* genus that causes pustular dermatitis. We identified a cluster of genes in PVNZ that encode three unique chemokine-binding proteins (CBPs) namely ORF112.0, ORF112.3 and ORF112.6. Chemokines are a large family of molecules that direct cell trafficking to sites of inflammation and through lymphatic organs. The PVNZ-CBPs were analyzed by surface plasmon resonance against a broad spectrum of CXC, CC, XC and CX_3_C chemokines and were found to differ in their specificity and binding affinity. ORF112.0 interacted with chemokines from the CXC, CC and XC classes of chemokines with nM affinities. The ORF112.3 showed a preference for CXC chemokines, while ORF112.6 showed pM affinity binding for CC chemokines. Structural modeling analysis showed alterations in the chemokine binding sites of the CBPs, although the core structure containing two ß-sheets and three α-helices being conserved with the other *parapoxvirus* CBPs. Chemotaxis assays using neutrophils and monocytes revealed inhibitory impact of the CBPs on cell migration. Our results suggest that the PVNZ-CBPs are likely to have evolved through a process of gene duplication and divergence, and may have a role in suppressing inflammation and the anti-viral immune response.

## Introduction

Parapoxviruses comprise a genus of the *Poxviridae* family (Mercer et al., 1997; Fleming and Mercer, 2007). The most well-known members are orf virus (ORFV), which predominantly infects sheep and goats, and bovine papular stomatitis virus (BPSV) and pseudocowpox virus (PCPV), which infect cattle (Fleming and Mercer, 2007). ORFV, BPSV, and PCPV are all zoonotic viruses and are distributed worldwide. Parapoxvirus infections of New Zealand farmed red deer (*Cervus elaphus*) were first reported in 1987 (Horner et al., 1987) and isolates were characterized by their oval shape and “ball of wool” appearance by electron microscopy. While ORFV has been reported to infect Japanese serow (Inoshima et al., 2001), reindeer (Falk, 1978; Klein and Tryland, 2005), and white-tailed deer (Smith et al., 1991; Kuhl et al., 2003; Roess et al., 2010), restriction endonuclease analysis of genomic DNA of the New Zealand red deer isolates revealed that it was a unique parapoxvirus species and was named Parapoxvirus of red deer in New Zealand (PVNZ) (Robinson and Mercer, 1995). Since its discovery in New Zealand, the virus has been isolated from red deer in Europe (Scagliarini et al., 2011; Friederichs et al., 2015). Restriction fragment length polymorphisms revealed a strong match of the European isolates with PVNZ, and like PVNZ, there were clear differences from other members of the genus. It is thought that PVNZ was most likely introduced into New Zealand in the early part of the 20th century from infected red deer introduced from the United Kingdom and Europe (Friederichs et al., 2015).

In common with other established members of the *Parapoxvirus* genus, PVNZ infects skin causing pustular dermatitis and severe proliferative lesions, erosions and ulcers on the lips and hard palate have been reported (Horner et al., 1987; Robinson and Mercer, 1995; Scagliarini et al., 2011). Skin lesions induced by PVNZ are inflamed and like other parapoxvirus infections, contain a dense mononuclear dermal infiltrate of cells such as neutrophils, macrophages and lymphocytes (Horner et al., 1987; Scagliarini et al., 2011; Friederichs et al., 2015). Infections of the mucosal epithelium is known to seriously debilitate the ability of animals to feed with deaths reported (Scagliarini et al., 2011), whilst infections of red deer antler velvet are of economic importance (Horner et al., 1987). PVNZ infections usually resolve in 6–8 weeks. The virus has been isolated from tonsils of asymptomatic deer (0.7%) and it is thought the virus may persist in a small number of animals within the population (Friederichs et al., 2015).

Parapoxviruses encode a number of secreted factors involved in immune evasion and angiogenesis. These factors include an interleukin-10 (IL-10) like molecule (Fleming et al., 1997; Imlach et al., 2002; Lateef et al., 2003), a vascular endothelial growth factor (VEGF) (Lyttle et al., 1994; Wise et al., 1999; Savory et al., 2000; Friederichs et al., 2015), a granulocyte macrophage colony stimulating factor (GM-CSF) and IL-2 inhibitory factor (GIF) (Deane et al., 2000) and chemokine-binding proteins (CBPs) (Seet et al., 2003a; Lateef et al., 2009; Sharif et al., 2016). Evidence suggests that the immunomodulators of ORFV are involved in dampening inflammation and inhibiting the host’s innate defenses so as to allow the virus to reinfect its host (Fleming et al., 2015).

Chemokines are a large family of molecules that direct cell trafficking within mammalian species. They do this by forming chemokine gradients on endothelial surfaces through their glycosaminoglycan binding domains. G-protein coupled receptors on leukocytes bind chemokines initiating chemotaxis in the direction of the gradient. Chemokines are functionally divided into two groups, inflammatory and homeostatic and have also been classified according to the number and spatial arrangement of cysteine residues at the N-terminus. The largest classes are the CXC and CC chemokines, while the XC and CX_3_C chemokines have fewer members (Janeway et al., 1999; Rossi and Zlotnik, 2000). Inflammatory chemokines such as CCL2, CCL3, CCL5 CXCL1, CXCL2, and XCL1 are produced at high concentrations during injury or infection and are primarily involved in recruiting inflammatory cells such as neutrophils, monocytes, dendritic cells (DC), mast cells and T-lymphocytes from the blood or nearby tissue to the inflamed site. Homeostatic chemokines are produced constitutively from the thymus or lymphoid tissue and direct the movement of cells, in particular antigen-presenting cells, through lymphoid organs to initiate the adaptive responses (Rossi and Zlotnik, 2000).

Chemokine-binding proteins that have been characterized for ORFV and BPSV are related to the CC chemokine inhibitory (CCI) proteins identified for members of the *Orthopoxvirus* and *Leporipoxvirus* genera. Despite relatively low sequence homology (12–18% identity and 26–32% similarity) between the CBPs and CCIs, these proteins share notable regions of identity, six cysteine residues that form three disulfide bonds, and a common structural core (Seet et al., 2003a). The major differences between the CBPs and CCIs lie in their binding spectrum. Recent studies show that the parapoxvirus CBPs are broad-spectrum chemokine binders, as ORFV and BPSV CBPs bind across the CXC, CC, and XC classes with pM affinity (Seet et al., 2003a; Lateef et al., 2009; Sharif et al., 2016). In contrast, the CCIs are more restricted in their ability to bind chemokines and only bind members of the CC class (Seet et al., 2003b).

The crystal structure of ORFV-CBP alone and in complex with chemokines has been solved previously. The ORFV-CBP is aß-sheet sandwich composed of three α-helices and two ß-sheets. ß-sheet I is positively charged while ß-sheet II is negatively charged to bind chemokine binding sites. The binding of chemokines with ORFV-CBP involves the interaction of the N-terminal signaling domain of chemokine with a hydrophobic pocket on the CBP, and also a polar interaction between the positively charged 20s loop of chemokine and a broad negative groove on the ß-sheet II surface (Couanago et al., 2015).

We have identified a cluster of three genes in PVNZ that encode distinct CBPs. The proteins encoded by the genes differ in their structural details and chemokine-binding spectrum. The study suggests that the CBP genes have been duplicated from an early parapoxvirus ancestral gene and have evolved divergent ligand specificities that together bind members across the CXC, CC, and XC chemokine classes.

## Materials and Methods

### Virus Propagation

The PVNZ strain RD86 was a gift from Prof Bryce Buddle (AgResearch, Upper Hutt, New Zealand) and was isolated from lesions on the velvet of farmed red deer in New Zealand (Robinson and Mercer, 1995). The virus was propagated in primary bovine testis (BT) cells as described previously (Robinson et al., 1982; Robinson and Mercer, 1995). The BT cells were cultured in Eagles minimum essential medium (MEM) (GIBCO, Invitrogen) supplemented with 10% FBS, 5% lactalbumen hydrolyzate and PKS solution [kanamycin, (Roche Life Science); streptomycin-penicillin (Gibco)] and incubated at 37°C in a humidified 7% CO_2_ atmosphere. The viral particles were purified and DNA extracted as described previously (Ueda et al., 2007).

### Cloning, Expression, and Purification of CBPs

The CBP open reading frames were amplified by PCR using plasmid PVU550 as a template (constructed by cloning the BamHI-L restriction endonuclease fragment of PVNZ genomic DNA into pTZ18R) followed by DNA sequencing, (Ueda et al., 2007) with the following primers: 112.0PVNZ_F (5′-AAGGCGCGCCTGAGGTGTCTGATACTCACG -3′) and 112.0PVNZ_R (5′-GGGGCGCGCCTGAACATCAAATGTCTCGTTTCC-3′), 112.3PVNZ_F (5′-CCGGCGCGCCTGAAGAGATTCATATTTGCGGCGCTATG-3′) and 112.3PVNZ_R (5′-AAGGCGCGCCTGAGGATCAAGTTCCTCGTCGTC-3′), 112.6PVNZ_F (5′-ACGGCGCGCCTGAAGACGCTCTTGTTAGCAGC-3′) and 112.6PVNZ_R (5′-AAGGCGCGCCTGCTCGCTCAGTTCGCTGAC-3′). The PCR products were digested with AscI and ligated into a pAPEX-3-derived vector. The recombinant CBPs, tagged with the FLAG octapeptide at the C terminus, were expressed in 293-EBNA cells, then purified and quantified, as previously described (Inder et al., 2007).

### Sequence and Structural Analysis

Sequence alignment and phylogenetic analysis were performed using of ClustalW alignment (DNASTAR version 10.0.1) based on available sequences at the National Centre for Biotechnology Information (NCBI) database^1^. The structural analysis was performed based on crystal structure of ORFV-CBP strain NZ2 alone (4P5I) and in complex with CCL2 (4ZK9) using Fold and Function Assignment System (FFAS) server available at http://ffas.godziklab.org Predicted structural elements and sites of glycosylation, dimerization and binding interactions were determined and visualized using PyMOL platform^2^.

### Surface Plasmon Resonance Assay

Prior to performing the surface plasmon resonance (SPR) assay, the viral CBP was dialyzed in HBS-EP buffer (20 mM HEPES, 150 mM NaCl, 3.4 mM EDTA, 0.005% Polysorbate 20, pH 7.4) using 12–14 Da Spectra/Por^®^ 2 Standard Grade Regenerated Cellulose membrane (Spectrum Lab Inc.). All SPR experiments were performed at 25°C using a Biacore X100 instrument (Biacore) as described previously (Sharif et al., 2016). Each CBP was immobilized on a separate CM5 sensor chip (Biacore) at 300–400 response units (pg/mm^2^) in 10 mM sodium acetate pH 4.5 by standard amine coupling method. Recombinant mouse chemokines (R&D Systems, listed in Table 1) were reconstituted, and serial dilutions in HBS-EP buffer run over the CM5 chips in triplicate for 3 min. The chemokines were allowed to dissociate for 10 min and then the sensor chips were regenerated by injecting 10 mM Glycine pH 2.0 (GE Healthcare). The sensorgrams produced were globally fitted with a 1:1 binding model and used for kinetics analysis by BIAevaluation software (version 2.0.1 Biacore).

**Table 1:** Binding profiles of PVNZ-CBPs.

Chemokines	PVNZ112.0-CBP			PVNZ112.3-CBP			PVNZ112.6-CBP		
	*k*_a_ (× 10^6^M^-1^s^-1^)	*k_d_* (× 10^-3^s^-1^)	*K*_D_ (nM)	*k_a_* (× 10^6^M^-1^s^-1^)	*k*_d_ (× 10^-3^s^-1^)	*K*_D_ (nM)	*k_a_* (× 10^6^M^-1^s^-1^)	*k_d_* (× 10^-3^s^-1^)	*K*_D_ (nM)
CXCL1	NB	NB	NB	2.54 ± 0.02	1.69 ± 0.02	0.66	NM	NM	NM
CXCL2	NB	NB	NB	1.79 ± 0.04	0.87 ± 0.02	0.48	NM	NM	NM
CXCL4	0.17 ± 0.006	4.40 ± 0.03	25.88	1.29 ± 0.06	1.26 ± 0.03	0.97	18.5 ± 3.69	11.03 ± 1.08	0.59
CXCL10	0.97 ± 0.02	7.08 ± 0.08	7.29	0.24 ± 0.003	19.8 ± 0.2	82.50	0.14 ± 0.004	1.46 ± 0.02	10.42
CXCL12	NM	NM	NM	NM	NM	NM	NM	NM	NM
CCL2	NB	NB	NB	NB	NB	NB	11.8 ± 2.35	0.37 ± 0.005	0.03
CCL3	NB	NB	NB	NB	NB	NB	14.22 ± 1.47	35.81 ± 5.44	2.51
CCL5	NM	NM	>230	1.65 ± 0.04	52.05 ± 0.6	31.54	27.46 ± 4.08	47.94 ± 6.06	1.74
CCL19	NM	NM	NM	0.01 ± 0.0007	1.58 ± 0.02	15.80	10.01 ± 1.36	1.32 ± 0.06	0.13
CCL21	0.53 ± 0.004	6.82 ± 0.03	12.86	0.14 ± 0.006	2.51 ± 0.01	17.92	19.51 ± 3.02	10.3 ± 2.08	0.52
CCL22	NM	NM	NM	NB	NB	NB	21.7 ± 3.4	0.47 ± 0.005	0.02
XCL1	0.06 ± 0.0002	2.03 ± 0.02	30.83	NM	NM	NM	NM	NM	NM
CX_3_CL1	NB	NB	NB	NM	NM	NM	NB	NB	NB

*The binding affinities of CBPs against various murine chemokines were determined using SPR assay. NB, no binding; NM, non-measurable binding.*

### Cells and Transwell Migration Assay

Murine promyelocyte (MPRO) cells were purchased from the American Type Culture Collection (ATCC CRL-11422, clone 2.1) and cultured in Iscove’s modified Dulbecco medium (IMDM, Gibco) supplemented with heat-inactivated 20% horse serum (Gibco), 10 ng/ml GM-CSF (R&D Systems), PSK, and 50 μg/L gentamycin sulphate (AppliChem) as described previously (Sharif et al., 2016). The cells were incubated at 37°C in a humidified incubator with 5% CO_2_ (Panasonic), and sub-cultured every two days to maintain the cell density at 4–5 × 10^5^ cells/ml.

Mature neutrophils were derived from MPRO cells, which have been reported as a valid model for producing functionally active murine neutrophils (Gaines and Berliner, 2005). Differentiation of MPRO cells to mature neutrophils was induced with 10 μM all-*trans* retinoic acid (ATRA, Sigma) in complete IMDM growth medium in the dark for three days. Morphologic maturation of neutrophils was confirmed by Giemsa May-Grunwald staining (Sigma) of cytospins under light microscopy and mature neutrophils were characterized by FACS as described previously (Sharif et al., 2016).

The human monocyte cell line THP-1 (ATCC^®^ TIB-202TM) was cultured in RPMI-1640 medium supplemented with FBS at 10% for growth and 5% for culture maintenance. The cells were incubated at 37°C with 5% CO_2_ and were re-suspended in fresh media every two days to maintain the cell density between 5–8 × 10^5^ cells/ml.

The transwell migration assays were performed using 6.5 mm-diameter Corning Transwell 24-well plates (Corning Life Sciences) with either 3 μm (for neutrophils) or 5 μm (for monocytes) pore size polycarbonate membranes. All transwell assays were performed in duplicate with control wells. Prior to each assay, transwell plates were pre-equilibrated with 1 ml of transwell medium (growth medium with 1% BSA) at 37°C with 5% CO_2_ for 1 h. Neutrophils (5 × 10^5^) or monocytes (1 × 10^5^) suspended in 100 μl transwell medium were carefully placed into the inserts, and the plates were incubated at 37°C with 5% CO_2_ (Panasonic). The incubation times for neutrophil and monocyte transmigration were 2 and 3 h, respectively (Lateef et al., 2009; Sharif et al., 2016). In neutrophil migration assays, transmigrated cells were collected from the lower chambers and counted simultaneously with a volume of 30 μl of AccouCount fluorescent particles (ACFP) (Spherotech) on a flow cytometer (FACSFortessa, BD) for 2 min (Sharif et al., 2016). In monocyte migration assays, the transmigrated cells were collected from the underside of the transwell membrane, stained and enumerated (20). The data was analyzed for significant differences between treatments (*P* < 0.05) using ANOVA and a *post hoc* Tukey’s test using GraphPad Prism.

## Results

### PVNZ Encodes Three Putative Chemokine-Binding Proteins

**FIGURE 1:**
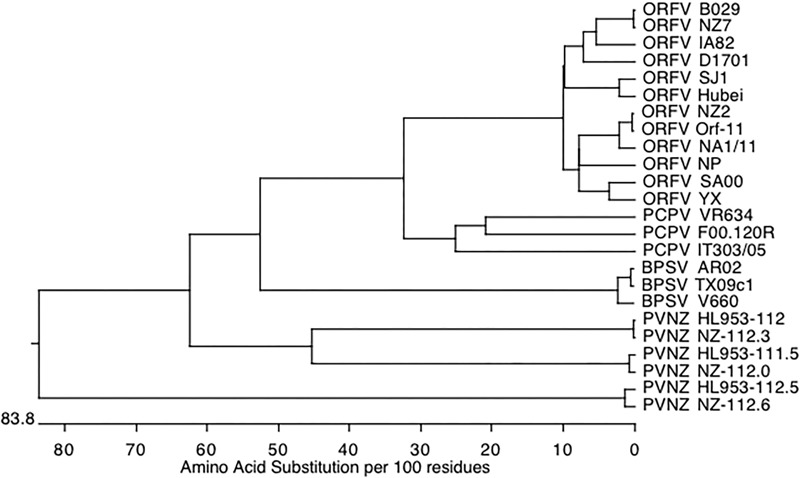
Phylogenetic analysis of CBPs encoded by parapoxviruses. The phylogenetic tree was generated by using ClustalW alignment (DNASTAR version 10.0.1) and shows the relatedness of the CBPs among PPVs.

Genomic sequencing of PVNZ revealed a cluster of three ORFs *ORF112.0, ORF112.3*, and *ORF112.6* near the right end of the genome (GenBank accession numbers MK947455, MK947456, MK947457, respectively). The ORFs show homology to the ORFV and BPSV CBPs and display typical early poxvirus promoter sequences and transcription termination sequences (T_5_NT) downstream of the coding sequence. The putative CBPs differ in size with *ORF112.0, ORF112.3*, and *ORF112.6* encoding proteins of 276, 277, and 292 amino acids (AA), respectively, with predicted masses of 30,753, 31,371 and 32,790 Da, respectively (Supplementary Figure S1). The AA sequences of PVNZ-CBP 112.0, 112.3, and 112.6 were identical (99.3 to 100% identity) to three ORFs of a reference parapoxvirus genome, however they have been named differently as 111.5, 112, and 112.5 in the previous study (Friederichs et al., 2015). Clustal alignment showed that the CBP112.0 and CBP112.3 polypeptides share high similarity with an AA identity of 41.9% whereas these polypeptides are remarkably dissimilar to CBP112.6 with 24.2 and 24.1% identity, respectively. The CBP sequences of PVNZ were also aligned with the other parapoxvirus CBPs available in the GenBank database, and shown as a pairwise comparison table (Supplementary Table S1). Phylogenetic analysis showed a species-based CBP clustering where the PVNZ-CBPs, in particular PVNZ112.6, are considerably different from all other parapoxvirus CBPs (Figure 1). It appears that the PVNZ CBPs have arisen from a common ancestral parapoxvirus gene that has undergone duplication and divergence within the PVNZ lineage.

### The PVNZ-CBPs Display Diverse Chemokine-Binding Specificity

The similarity of the PVNZ putative CBPs with other well-characterized parapoxvirus CBPs, suggested that some or all of the expressed proteins would interact with members of the chemokine family. To assess interactions with chemokines across all classes we performed a binding analysis using SPR assay. The CBP-FLAG proteins were coupled to CM5 chips, exposed to thirteen murine chemokines from four different classes, and monitored as described previously (Sharif et al., 2016). The results of interactions are summarized in Table 1.

Surface plasmon resonance analysis of PVNZ112.0-CBP showed that it bound to certain chemokines from different classes with nM affinity including CXCL4 and CXCL10 (*K_D_* = 25.8 and 7.2 nM, respectively), and also CCL21 (*K_D_* = 12.8 nM) and XCL1 (*K_D_* = 30.8 nM) (Supplementary Figure S2). This CBP either did not bind to the rest of the chemokines tested (CXCL1, CXCL2, CCL2, CCL3, and CX3CL1) or showed an extremely weak binding with kinetics constants beyond the limitations of the machine and thus are presented as non-measurable binding (CXCL12, CCL5, CCL19, and CCL22) in Table 1.

PVNZ112.3-CBP showed a preference for binding CXC chemokines with binding affinities in a sub nanomolar range. The CBP bound to CXCL1, CXCL2, and CXCL4 with strong binding affinity (*K_D_* = 0.486 - 0.976 nM) (Supplementary Figure S3). Interestingly, the CXCL10 chemokine showed a weaker binding than others (*K_D_* = 82.5 nM) with a very fast dissociation rate (*k*_d_ = 19.8 × 10^-3^ s^-1^) that has two orders magnitude difference with others (Supplementary Figure S3). In addition, the PVNZ112.3-CBP binds CC chemokines selectively with nM binding affinity to CCL5, CCL19, and CCL21 (*K_D_* = 15.8–31.5 nM) (Supplementary Figure S4). Further, PVNZ112.3 showed no binding to CCL2, CCL3, and CCL22 nor did it bind XCL1 and CX_3_CL1.

In general, the SPR results showed that PVNZ112.6-CBP is a CC chemokine blocker with strong binding affinity for several members of this class. The chemokines that showed very strong binding affinity were CCL2 (Supplementary Figure S5), CCL19 and CCL22 (Supplementary Figure S6) with *K_D_* values of 21.65, 31.3 and 131 pM, respectively. Slightly lower binding affinity was found for CCL21, CCL5 (Supplementary Figure S6) and CCL3 (*K_D_* = 0.52–2.5 nM) (Supplementary Figure S5). PVNZ112.6-CBP also bound to CXCL4 (*K*_D_ = 0.59 nM) and CXCL10 (*K*_D_ = 10.42 nM) (Supplementary Figure S5), but did not interact with XCL1 and CX_3_CL1.

### Structural Properties of the PVNZ CBPs

**FIGURE 2:**
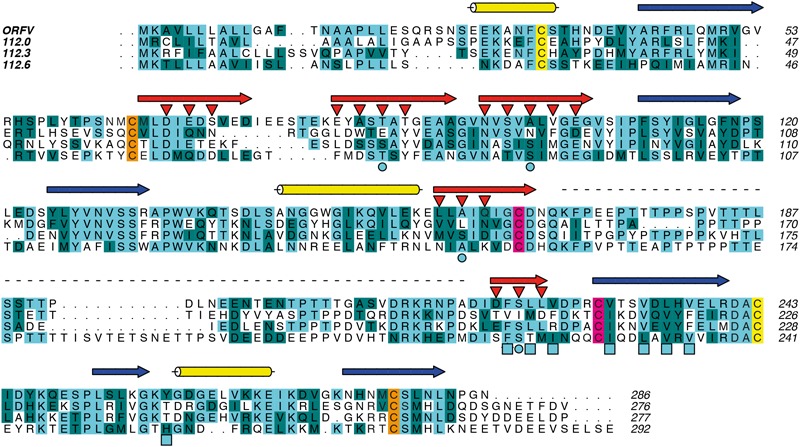
Structure-based sequence alignment of ORFV and PVNZ CBPs using FFAS. Predicted structural elements and sites of glycosylation, dimerization and binding interactions are shown. Identical and conserved amino acids are highlighted in light and dark blue, respectively. The conserved cysteines are shown in pair colors (yellow, orange and pink). The PVNZ-CBPs display a common β-sheet sandwich structure including three predicted α-helices (yellow cylinders) and two β-sheets. The strands of β-sheet I (blue arrows) are enriched with basic residues that might serve as a GAG-binding surface, while the strands of β-sheet II (red arrows) include acidic and hydrophobic residues predicted to be involved in chemokine-binding (red triangles). The light blue circles and squares indicate residues forming the N-loop anchoring region. The dashed line shows a distinct region in the connecting loop between β-strands 7 and 8. The numbers at right edge show ORFV residues.

To gain structural insights into the widely different ligand specificities of the PVNZ-CBPs, we used the ORFV-CBP whose crystal structure alone and in complex with chemokines has been solved (Couanago et al., 2015). The structure-based sequence alignment of PVNZ-CBPs suggests that although their sequences have only 30–37.5% identity to the ORFV-CBP, their core structure consisting of two ß-sheets and three α-helices is conserved (Figure 2). The clustal alignment shows small blocks of conserved sequence scattered throughout the polypeptides. Six cysteine residues that are conserved in other parapoxvirus CBPs are also conserved in the same relative position in the PVNZ-CBPs. Interestingly 112.0 and 112.3 each have an additional cysteine at the near N-terminus.

**FIGURE 3:**
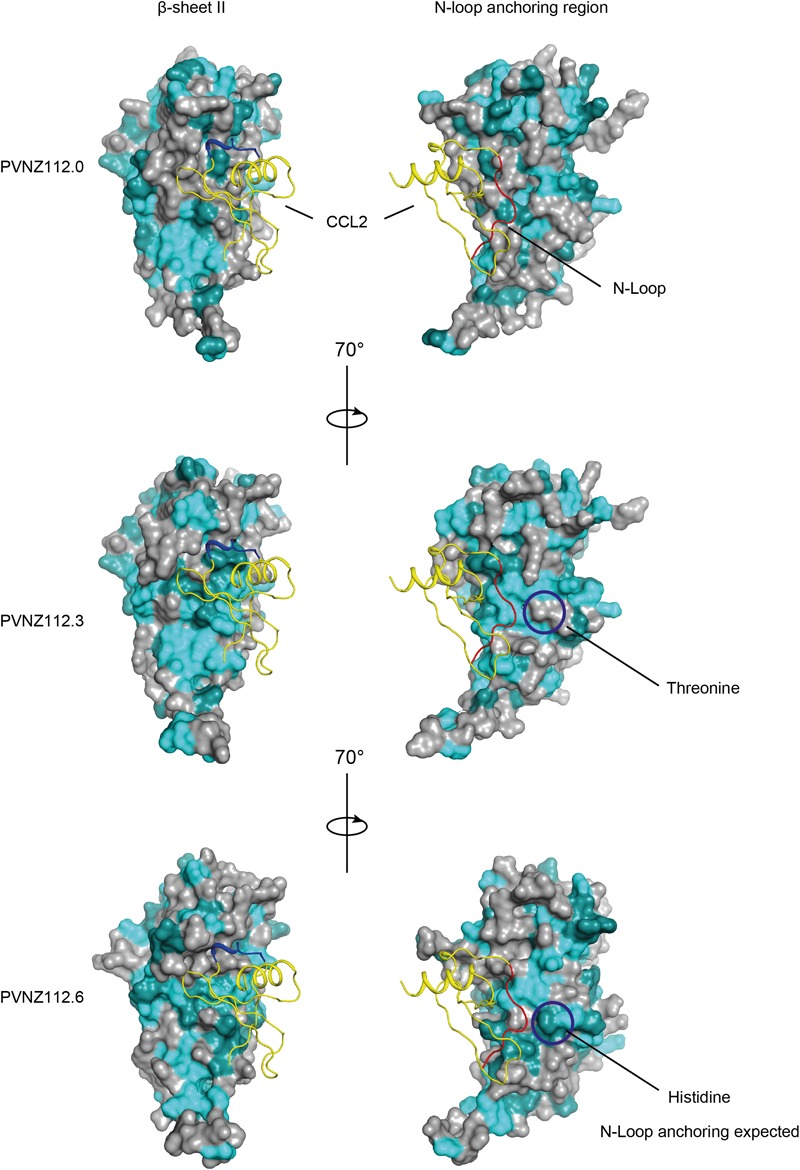
Homology models of PVNZ-CBPs in potential binding to CCL2 chemokine (PDB: 4ZK9) using FFAS and PyMOL. The CBPs show the common core structure of the ORFV-CBP with some modifications on the binding sites as indicated by circles. The color scheme is identical to Figure 2. The CCL2 chemokine is shown as a yellow ribbon with highlighted binding sites 20s loop (blue) and N-loop (red). Some substitutions at the N-loop anchoring region of PVNZ112.3-CBP (e.g., tyrosine to threonine) and PVNZ112.6-CBP (e.g., tyrosine to histidine) could affect the geometry of the CBPs and their binding affinity to some chemokines.

**FIGURE 4:**
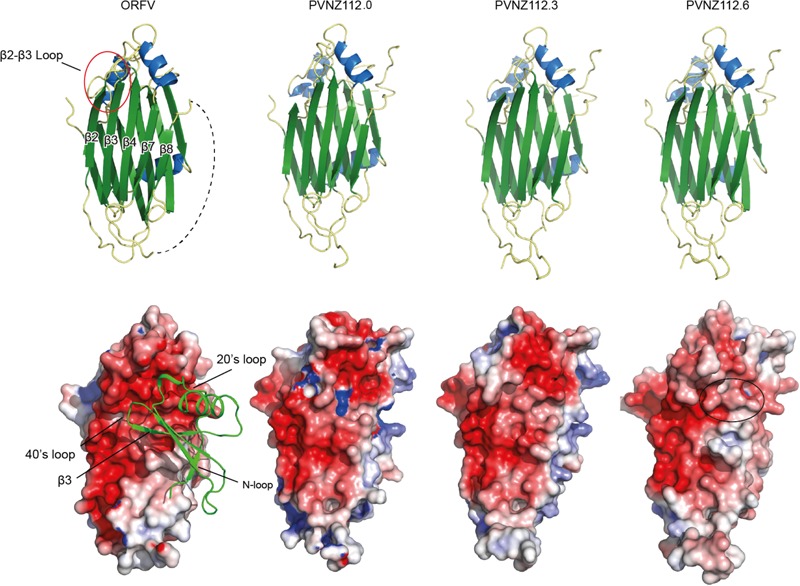
Structure of ORFV and PVNZ CBPs homology models. The top panel shows the structural elements where helices and strands are shown in blue and green, respectively. The variable connecting loop between β-strands 2 and 3 is red-circled on the ORFV-CBP structure. The disordered loop connecting β7-β8 is shown in dashed line. The bottom panel shows the electrostatic surface of the CBPs (red – negative and blue – positive charge; PyMOL). The CCL2 molecule (green ribbon) is shown in the ORFV-CBP structure to illustrate binding to CCL2. Of note, the PVNZ112.0-CBP elucidates positively charged areas that are not favorable for binding positive chemokines. The black circle on the PVNZ112.6 structure also shows slightly decreased negative charge compared to that of ORFV-CBP.

Mapping of the conserved residues of PVNZ-CBPs onto the ORFV-CBP crystal structure provided molecular insights into the binding properties of the PVNZ CBPs (Figure 3). The PVNZ112.0-CBP sequence alignment showed that the two key regions, a hydrophobic pocket and a broad negative groove on the β-sheet II surface are least conserved which could affect chemokine-binding affinity of this CBP. Furthermore, the electrostatic analysis of the PVNZ112.0-CBP revealed positively charged areas that are not favorable for binding to positive-charged chemokines (Figure 4). The modeling data suggests that of the three PVNZ-CBPs, CBP-112.0 is most dissimilar to the known structure of ORFV-CBP and this divergence appears to be reflected in its relatively weak interactions with the chemokines tested by SPR assay (Table 1). PVNZ112.3-CBP maintains the surface of the β-sheet II structure similar to that of the ORFV-CBP. The N-loop anchoring region of this protein however, has a few major alterations, e.g., tyrosine to threonine substitution that could affect its geometry and reduce binding affinity of this CBP to some chemokines. Of the three PVNZ-CBPs, the residues in CBP-112.6 are most strongly conserved with ORFV-CBP and displays tight binding affinities, especially to CC chemokines. Molecular studies will provide further insight into how differences in the structure of PVNZ-CBPs relate to their chemokine binding preferences.

### The PVNZ-CBPs Inhibit Chemokine-Induced Cell Migration of Neutrophils and Monocytes

Parapoxvirus of red deer in New Zealand lesions have been described as proliferative dermatitis with moderate inflammatory infiltrate of neutrophils, lymphocytes and macrophages (Horner et al., 1987; Scagliarini et al., 2011). Neutrophils are among the first cells to respond to injury or infection and form the first line of defense against invading microorganisms. The recruited neutrophils secrete pro-inflammatory cytokines and chemokines that attract monocytes, DCs and lymphocytes to shape the immune response (Janeway et al., 1999). As the three CBPs expressed by PVNZ together bind a broad range of inflammatory chemokines, we performed functional assays to test their ability to inhibit neutrophil and monocyte chemotaxis using a transwell migration system. The assay is based on the ability of CBPs to occlude the receptor-binding site of the chemokine and thus prevent it from binding to its cognate receptor and inducing cell migration. We tested the ability of the CBPs to interact with a small range of chemokines from CXC, CC and XC classes that would differentiate their functional activities.

Neutrophils were derived from a differentiated MPRO cell line and their maturation, following stimulation with retinoic acid was confirmed by microscopic examination, with flow cytometric analysis confirming high levels of Ly-6G and CD11b expression with ∼80% expressing CXCR2 that are characteristic markers for mature neutrophils (Jaeger et al., 2012). A maximal 3–4-fold increase in cell migration was observed at 200 ng/ml of CXCL1, CXCL2, and XCL1 and 100 ng/ml of CCL3 (data not shown). The effect of the PVNZ-CBPs was then investigated by adding a titration of CBP to the above amount of chemokine in the transwell migration assay. Monocytes were generated from mouse bone marrow cell culture and expressed the common monocyte markers CD115 and CD11b with ∼75% Gr-1 positivity, characterizing inflammatory monocytes (Francke et al., 2011). Initial experiments showed a 3–4-fold increase in monocyte migration at 25 ng/ml of CCL3 (data not shown).

**FIGURE 5:**
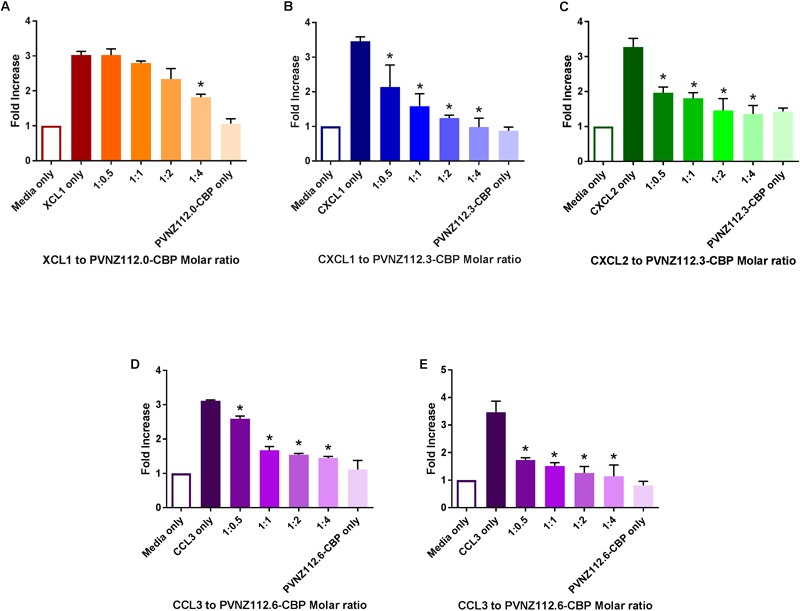
The inhibitory effect of PVNZ-CBPs on *in vitro* migration of neutrophils and monocytes in response to binding chemokines. Neutrophils (5 × 10^5^) or THP-1 monocytes (1 × 10^5^) were placed into the upper chamber of the transwell migration systems (A–D and E, respectively) containing 200 ng/ml of XCL1 **(A)**, CXCL1 **(B)**, CXCL2 **(C)** or 100 ng/ml of CCL3 **(D,E)** chemokines with or without serial dilutions of the PVNZ-CBPs to give the molar ratios shown (chemokine:CBP). The neutrophils and monocytes migration systems were incubated for 2 and 3 h, respectively. The transmigrated cells were collected and counted to calculate fold increase responses compared to the media-only control. The combined data are shown as mean ± SD of duplicate measures across three independent experiments with significant differences to chemokine-only indicated by asterisks (*P* < 0.05, ANOVA, Tukey‘s test).

The three PVNZ-CBPs were then tested for their ability to inhibit either neutrophil or monocyte migration in response to three classes of chemokines. We predicted that PVNZ112.0-CBP would bind XCL1 and inhibit neutrophil migration, whereas, PVNZ112.3-CBP and PVNZ112.6-CBP would have no effect on this chemokine in the transwell assay. The results showed that PVNZ112.0-CBP had significant inhibition of neutrophil migration in response to XCL1 when at 4-fold excess (Figure 5A) but did not inhibit migration of neutrophils or monocytes from other classes induced by either CXCL1, CXCL2 or CCL3 (data not shown). PVNZ112.3-CBP showed a preference for CXC chemokines and potently inhibited neutrophil migration induced by CXCL1 and CXCL2 when at half the amount of the chemokine (Figure 5B,C). PVNZ112.3-CBP did not however, inhibit cell migration induced by CCL3 (Supplementary Figures S7A,F) nor XCL1 (Supplementary Figure S7B). PVNZ112.6-CBP showed a strong binding to CCL3 chemokine and therefore a significant inhibitory effect on migration of neutrophils and monocytes when at half the chemokine amount (Figure 5D,E). PVNZ112.6-CBP did not inhibit the migration of neutrophils induced with XCL1, CXCL1 or CXCL2 (Supplementary Figures S7C–E).

## Discussion

This study shows that the parapoxvirus PVNZ encodes three functionally distinct chemokine-binding proteins. This is in marked contrast to other characterized parapoxvirus CBPs encoded by ORFV and BPSV each of which only encodes a single CBP gene (Seet et al., 2003a; Lateef et al., 2009; Sharif et al., 2016; Fleming et al., 2017). Altogether the binding spectrum of the three PVNZ-CBPs spans a broad range of chemokines from three chemokine classes suggesting they have a critical role in counteracting the host immune response.

The three PVNZ-CBP genes are clustered within a near terminal region of the genome and appear to have evolved through a process of gene duplication, followed by mutation divergence. This mechanism is not unusual amongst the poxviruses and such ligand specificity divergence is seen with other genes from members of the parapoxvirus genus. The GIF gene, encoded by ORFV, that binds GM-CSF and IL-2 is structurally related to ORFV CBP, shares a common ancestor, but displays a shift in ligand specificity (Deane et al., 2000; Seet et al., 2003a). Interestingly the CBP and GIF genes are not contiguous in ORFV and are separated by four unknown genes (Mercer et al., 2006). Another example of gene duplication within the poxvirus family are the ankyrin-repeat proteins, and in ORFV four of the five ankyrin-repeat genes cluster closely together, but their sequence and localization within virus-infected cells suggests they have different functions (Mercer et al., 2005; Sonnberg et al., 2008). The mechanism of gene duplication could involve a process of recombination and develop over the time.

The PVNZ-CBPs also displayed differences in specificity and binding affinity. PVNZ-CBP112.3 displayed strong *p*M binding to CXCL1 and CXCL2 to which the other PVNZ-CBPs did not bind. On the other hand, PVNZ-CBP112.6 bound CCL2, CCL3, and CCL22 to which the other PVNZ-CBPs did not bind. The SPR data also revealed that the PVNZ-CBPs bound common chemokines such as CXCL4, CXCL10, CCL5, and CCL22 but interestingly binding affinity was highly variable, most likely reflecting subtle differences within their chemokine-binding domains. The modeling data suggested that of the three PVNZ-CBPs, CBP112.0 is most dissimilar to the known structure of ORFV-CBP and this divergence appears to be reflected in its relatively weak interactions with the chemokines tested. PVNZ112.0 CBP did not bind any of the murine chemokines with pM affinity. In contrast, CBP112.3 showed higher structural similarity to ORFV-CBP and showed strong binding to CXC chemokines. Of the three PVNZ-CBPs, CBP-112.6 is most strongly conserved with ORFV-CBP and displays tight binding affinities, especially to CCL chemokines. Molecular studies will provide further insight into how differences in the structure of PVNZ-CBPs relate to their chemokine binding preferences.

The evolution of poxvirus-encoded CBPs is intriguing. Of all the poxvirus CBP genes that have been discovered, none of proteins encoded bear any resemblance to cellular chemokine receptors, suggesting that these genes have not been acquired from the host at least in recent evolutionary times. In contrast, virulence factors encoded by orthopoxviruses such as the IFN-gamma-like receptor and the TNF-like receptor bear strong similarity to host genes (Lalani and McFadden, 1997; Alejo et al., 2006; Xue et al., 2011) suggesting that they have been captured from their host. It is possible that the coding sequence for a progenitor poxvirus CBP gene was originally derived from another receptor captured from the host that has adapted to bind chemokines during the course of evolution. Interestingly, all the orthopoxvirus and myxoma virus CBPs are highly conserved at the AA level whereas, the parapoxvirus CBPs are more divergent (Seet et al., 2003a; Sharif et al., 2016). The CCI proteins encoded by the orthopoxviruses and leporipoxviruses contain many blocks of identical AA sequence, possess four disulphide covalent bonds and are secreted as monomers. In contrast the ORFV, BPSV, and PVNZ CBPs are all only moderately similar at the AA level to the CCI proteins. The ORFV and BPSV CBPs have less cysteine covalent bonds and are secreted as dimers as shown by multi-angle laser-light scattering size-exclusion chromatography (MALLS-SEC) analysis (Couanago et al., 2015; Sharif et al., 2016). We have not performed MALLS-SEC analysis to determine whether the PVNZ-CBPs are expressed as dimers and it could be investigated in future studies.

Nelson et al. (2015) have identified a conserved β-sandwich fold among four groups of apparently unrelated poxviral proteins and named it poxvirus immune evasion (PIE) domain superfamily. The PIE domain-containing proteins are nonessential for viral replication, and can bind to different ligands such as chemokines, GAGs, GM-CSF, IL-2, and MHC class I molecules (Nelson et al., 2015). It is likely that the poxvirus CBPs have all derived from a common PIE ancestor and evolved to manipulate their host chemokines. It is thought that the evolution of the parapoxviruses occurred at a very early stage of vertebrate poxvirus evolution (Babkin and Shchelkunov, 2006). It would appear that as the parapoxvirus species have evolved, so to have their immunomodulators, such as the chemokine-binding proteins and that to a significant degree, this evolution has occurred within the parapoxvirus lineage. Part of this diversity could be host driven, however, the chemokines from different mammalian species are remarkably conserved. We have not attempted to test the binding of parapoxvirus CBPs against their specific host chemokines, but we have shown that ORFV, BPSV, and PVNZ CBPs all bind with high affinity to non-host murine chemokines. This raises the question why the parapoxvirus CBPs are so divergent, at least with the primary structure and this is partly explained by conserved substitutions. In addition, 3-D modeling studies based on the known crystallographic structure of ORFV-CBP suggests that this divergence maybe more apparent at AA level and in fact the parapoxvirus CBPs are remarkably similar to each other, having a two ß-sheet sandwich structure and three α-helices. The SPR assay showed that the PVNZ112.0-CBP binds to different chemokines with relatively low affinity. It’s possible that like ORFV-GIF, it has adapted to bind other cytokines such as GM-CSF and IL-2 (Deane et al., 2000), or like A41 protein of Vaccinia virus, interact with a broad spectrum of chemokines with low affinity (Bahar et al., 2008). However, such additional binding capacity was not investigated in this study.

Both ORFV and BPSV CBPs have evolved diverse chemokine-binding specificities across three chemokine classes and have each done so within a single protein, that begs the question why PVNZ has evolved three unique CBPs. Together the three PVNZ-CBPs, bind a set of chemokines that is little different from those bound by ORFV and BPSV CBPs (Seet et al., 2003a; Lateef et al., 2009; Couanago et al., 2015; Sharif et al., 2016). However, there are some differences such as that all PVNZ-CBPs bind CXCL10, albeit with low affinity, whereas ORFV and BPSV CBPs do not bind this chemokine. Further, CXCL12, a chemoattractant for T-lymphocytes and monocytes, is not bound by any parapoxvirus CBPs. As stated above, most of our analyses have been performed with murine chemokines and may not be truly representative of host species for the parapoxviruses. Clearly the critical chemokines targeted are those associated with skin inflammation and immunity. Our data suggests that the three PVNZ-CBPs have evolved to target specific chemokine classes with high affinity binding and it may not be possible for a single PVNZ-CBP molecule to bind such a broad spectrum with high affinity. In addition, there may be greater molar amounts of secreted CBPs expressed from three genes than from a single gene.

Parapoxvirus of red deer in New Zealand infections of red deer have been mainly found associated with deer antler velvet and it is possible that infection of this tissue is important for viral survival. Deer antler velvet is highly vascularized which enables rapid growth of the antler. Associated with highly vascularized tissue is a potent inflammatory and immune response. It is possible that the multiple PVNZ-CBPs are required to dampen inflammation in such a potent immune environment. To understand the role of the PVNZ CBPs in virulence and pathogenesis in its natural host, recombinant viruses in which one or a combination of the genes are knocked out would need to be constructed for animal studies. The contribution of each gene to viral pathogenesis could then be assessed. Deletion of the broad-spectrum CBP gene of ORFV NZ2 severely attenuated virus infection in its natural host sheep, suggesting a critical role in subverting the host anti-viral response (Fleming et al., 2017). Future studies may reveal the importance of these divergent CBPs in viral pathogenesis in deer.

The characterization of CBPs encoded by PVNZ, illustrates further, the diversity of factors that have evolved in members of the parapoxvirus genus that target host immunity. Of particular significance is that parapoxviruses of red deer have evolved three structurally and functionally unique CBPs. Previously we and others have suggested that viral encoded CBPs may have potential as anti-inflammatory drugs (Lateef et al., 2010; Lee et al., 2015; Sharif et al., 2016). The discovery of a further three unique parapoxvirus CBPs adds to this potential resource.

## Data Availability

The raw data supporting the conclusions of this manuscript will be made available by the authors, without undue reservation, to any qualified researcher.

## Author Contributions

SS, SF, and ZL designed and conceived the experiments. SS, NU, YN, and SC performed the experiments. SS and SF wrote the manuscript. LW and AM assisted with the manuscript preparation and proofreading.

## Conflict of Interest Statement

The authors declare that the research was conducted in the absence of any commercial or financial relationships that could be construed as a potential conflict of interest.

## Footnotes

1 http://www.ncbi.nlm.nih.gov

2 http://www.pymol.org

## Supplementary Material

FIGURE S1 SDS-PAGE (left) and Western Blot (right) analysis of the PVNZ-CBPs using anti-FLAG antibody under reducing and non-reducing conditions. Coomassie blue staining shows protein bands of ∼40–45 kDa (reducing) corresponding to CBPs expressed by PVNZ112.0, PVNZ112.3, and PVNZ112.6.

FIGURE S2 SPR sensor grams illustrating the bindings of PVNZ112.0-CBP to mouse chemokines. Serial concentrations of the chemokines were injected in triplicate over the immobilized CBP on a CM5 chip for 180 s, and then allowed to dissociate over 600 s. The obtained curves were globally fitted with BIAevaluation 3.2 software using a 1:1 binding model, and used for kinetics analysis presented in Supplementary Table S1.

FIGURES S3 AND S4 SPR sensor grams illustrating the bindings of PVNZ112.3-CBP to mouse chemokines. Serial concentrations of the chemokines were injected in triplicate over the immobilized CBP on a CM5 chip for 180 s, and then allowed to dissociate over 600 s. The obtained curves were globally fitted with BIAevaluation 3.2 software using a 1:1 binding model, and used for kinetics analysis presented in Supplementary Table S1.

FIGURES S5 AND S6 SPR sensor grams illustrating the bindings of PVNZ112.6-CBP to mouse chemokines. Serial concentrations of the chemokines were injected in triplicate over the immobilized CBP on a CM5 chip for 180 s, and then allowed to dissociate over 600 s. The obtained curves were globally fitted with BIAevaluation 3.2 software using a 1:1 binding model, and used for kinetics analysis presented in Supplementary Table S1.

FIGURE S7 PVNZ-CBPs show no effect on *in vitro* migration of neutrophils and monocytes in response to non-binding chemokines. Neutrophils (5 × 10^5^) or THP-1 monocytes (1 × 10^5^) were placed into the upper chamber of the transwell migration systems (**A–F**, respectively) containing 100 ng/ml of CCL3 **(A,F)**, 200 ng/ml of XCL1 **(B,C)**, CXCL1 **(D)** or CXCL2 **(E)** chemokines with or without serial dilutions of the PVNZ-CBPs to give the molar ratios shown (chemokine:CBP). The neutrophil and monocyte migration systems were incubated for 2 and 3 h, respectively. The transmigrated cells were collected and counted to calculate fold increase responses compared to the media-only control. The combined data are shown as mean ± SD of duplicate measures across three independent experiments. No significant differences to chemokine-only were observed (*P* > 0.05, ANOVA).

TABLE S1 Pairwise sequence comparison between CBPs encoded by parapoxviruses using MegAlign (ClustalW, DNASTAR version 10.0.1). Divergence (below diagonal) is calculated by comparing sequence pairs in units in relation to the phylogeny reconstructed by MegAlign. Percent identity (above diagonal) compares sequences directly, without accounting for phylogenetic relationships. Divergence is not usually the inverse of percent identity.
