# Supplementary figures and images for: Chemokine-Binding Proteins Encoded by Parapoxvirus of Red Deer of New Zealand Display Evidence of Gene Duplication and Divergence of Ligand Specificity
Source: Front Microbiol. 2019 Jun 25;10:1421. doi: 10.3389/fmicb.2019.01421 (PMC6603201; doi:10.3389/fmicb.2019.01421)

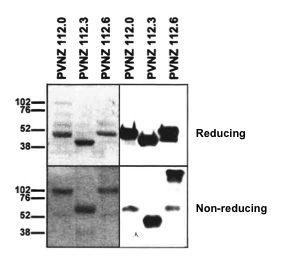

Supplement: FIGURE S1 — SDS-PAGE (left) and Western Blot (right) analysis of the PVNZ-CBPs using anti-FLAG antibody under reducing and non-reducing conditions. Coomassie blue staining shows protein bands of ∼40–45 kDa (reducing) corresponding to CBPs expressed by PVNZ112.0, PVNZ112.3, and PVNZ112.6. [file Image_1.tiff]

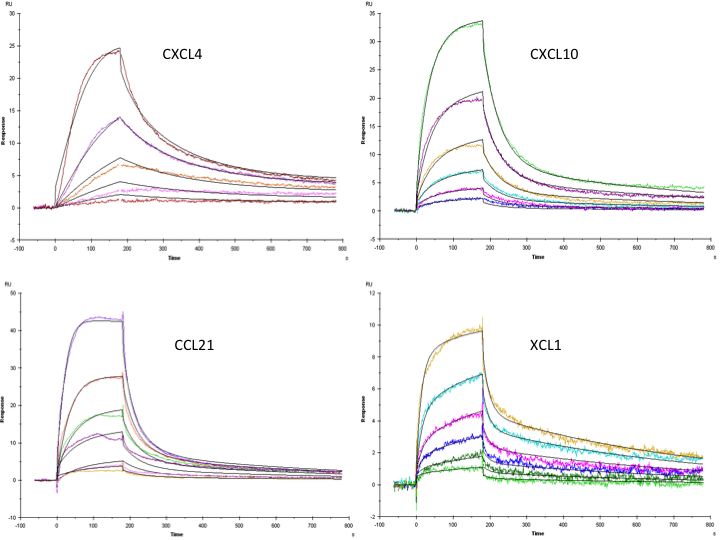

Supplement: FIGURE S2 — SPR sensor grams illustrating the bindings of PVNZ112.0-CBP to mouse chemokines. Serial concentrations of the chemokines were injected in triplicate over the immobilized CBP on a CM5 chip for 180 s, and then allowed to dissociate over 600 s. The obtained curves were globally fitted with BIAevaluation 3.2 software using a 1:1 binding model, and used for kinetics analysis presented in Supplementary Table S1. [file Image_2.TIFF]

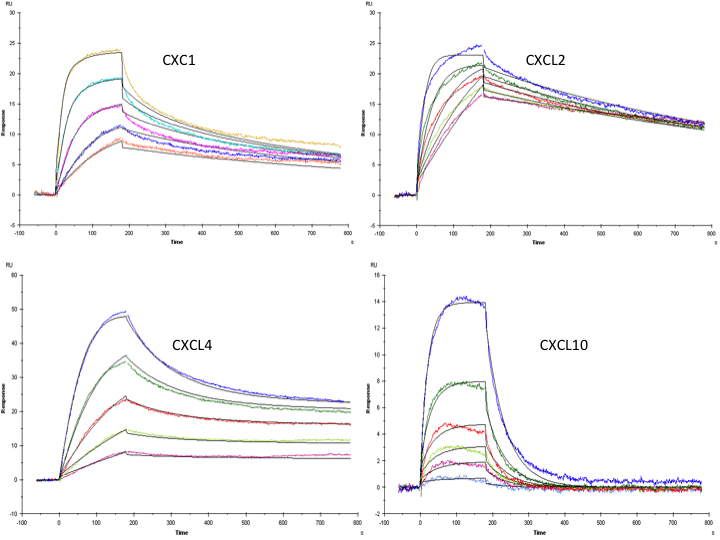

Supplement: FIGURES S3 AND S4 — SPR sensor grams illustrating the bindings of PVNZ112.3-CBP to mouse chemokines. Serial concentrations of the chemokines were injected in triplicate over the immobilized CBP on a CM5 chip for 180 s, and then allowed to dissociate over 600 s. The obtained curves were globally fitted with BIAevaluation 3.2 software using a 1:1 binding model, and used for kinetics analysis presented in Supplementary Table S1. [file Image_3.TIFF]

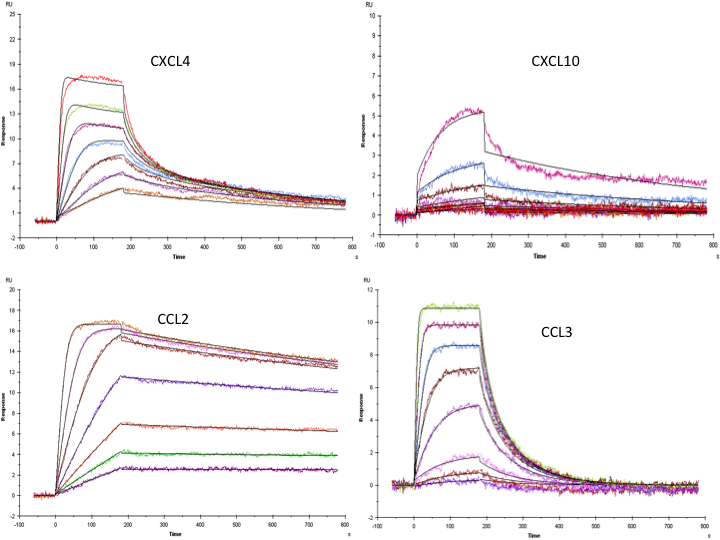

Supplement: FIGURES S5 AND S6 — SPR sensor grams illustrating the bindings of PVNZ112.6-CBP to mouse chemokines. Serial concentrations of the chemokines were injected in triplicate over the immobilized CBP on a CM5 chip for 180 s, and then allowed to dissociate over 600 s. The obtained curves were globally fitted with BIAevaluation 3.2 software using a 1:1 binding model, and used for kinetics analysis presented in Supplementary Table S1. [file Image_5.TIFF]

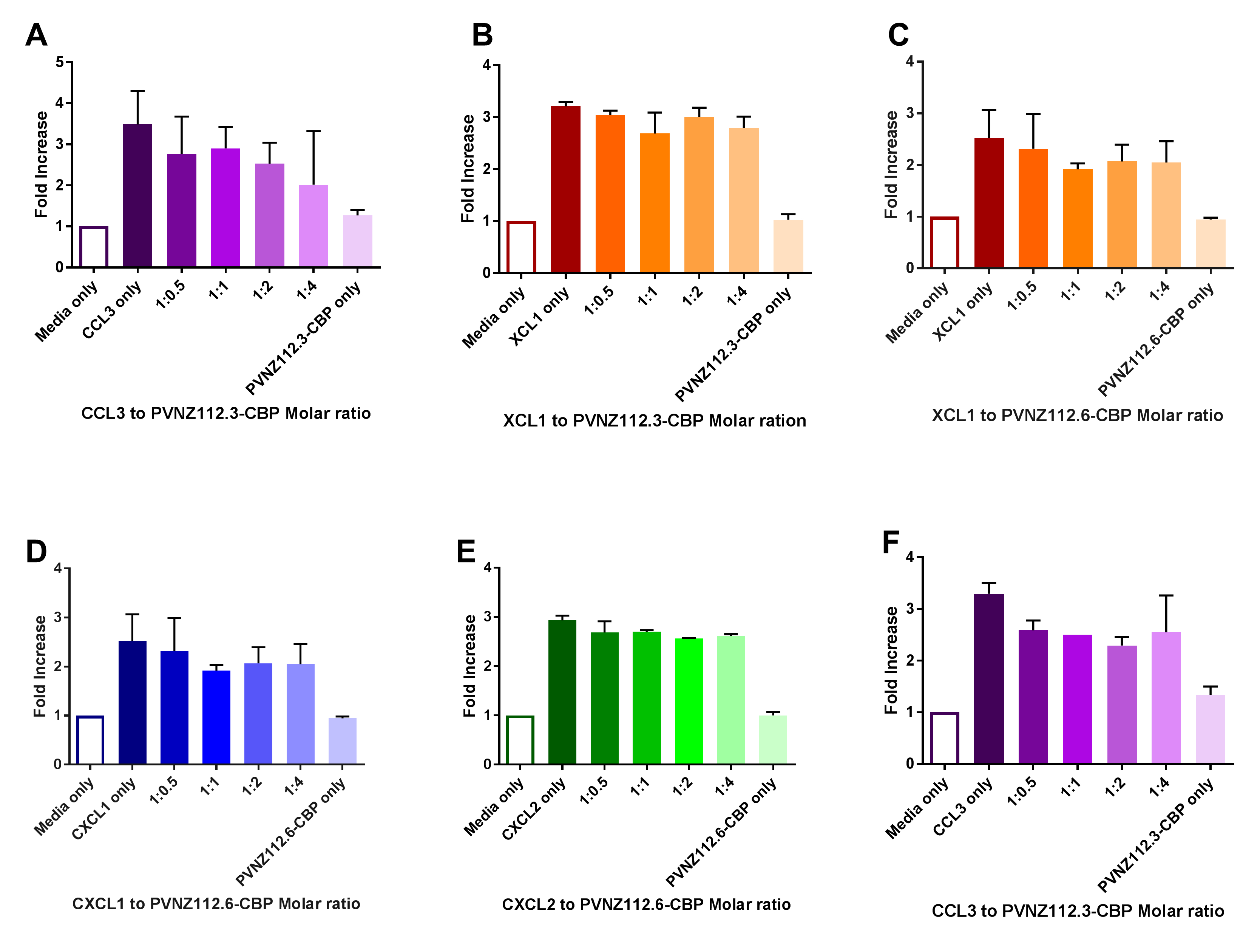

Supplement: FIGURE S7 — PVNZ-CBPs show no effect on in vitro migration of neutrophils and monocytes in response to non-binding chemokines. Neutrophils (5 × 10^5^) or THP-1 monocytes (1 × 10^5^) were placed into the upper chamber of the transwell migration systems (A–F, respectively) containing 100 ng/ml of CCL3 (A,F), 200 ng/ml of XCL1 (B,C), CXCL1 (D) or CXCL2 (E) chemokines with or without serial dilutions of the PVNZ-CBPs to give the molar ratios shown (chemokine:CBP). The neutrophil and monocyte migration systems were incubated for 2 and 3 h, respectively. The transmigrated cells were collected and counted to calculate fold increase responses compared to the media-only control. The combined data are shown as mean ± SD of duplicate measures across three independent experiments. No significant differences to chemokine-only were observed (P > 0.05, ANOVA). [file Image_7.JPEG]

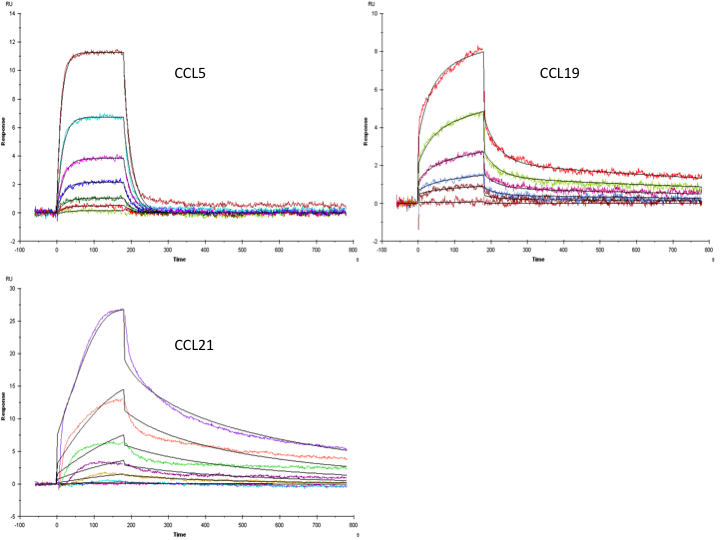

Supplement: Supplementary file 7 [file Image_4.TIFF]
