# Supplementary material for: Chemokine-Binding Proteins Encoded by Parapoxvirus of Red Deer of New Zealand Display Evidence of Gene Duplication and Divergence of Ligand Specificity
Source: Front Microbiol. 2019 Jun 25;10:1421. doi: 10.3389/fmicb.2019.01421 (PMC6603201; doi:10.3389/fmicb.2019.01421)
Supplement: TABLE S1 — Pairwise sequence comparison between CBPs encoded by parapoxviruses using MegAlign (ClustalW, DNASTAR version 10.0.1). Divergence (below diagonal) is calculated by comparing sequence pairs in units in relation to the phylogeny reconstructed by MegAlign. Percent identity (above diagonal) compares sequences directly, without accounting for phylogenetic relationships. Divergence is not usually the inverse of percent identity. [file Data_Sheet_1.PDF]

Percent Identity

Divergence

	1	2	3	4	5	6	7	8	9	10	11	12	13	14	15	16	17	18	19	20	21	22	23	24		
**1**		95.3	95.6	43.7	40.5	40.1	40.9	43.7	41.0	41.2	40.6	43.3	40.6	40.5	39.5	38.8	42.8	40.0	39.2	43.5	30.1	38.7	40.4	29.7	**1**	BPSV V660.pro
**2**	4.9		99.0	43.9	40.3	39.9	41.8	43.5	41.5	41.0	41.5	43.1	41.5	39.8	40.4	40.1	42.6	40.5	36.6	44.2	29.9	36.1	39.6	29.5	**2**	BPSV AR02.pro
**3**	4.5	1.0		43.7	40.1	39.8	41.7	43.3	41.4	40.8	41.3	43.0	41.3	39.8	40.2	39.9	42.4	40.4	37.3	44.0	30.1	36.8	39.6	29.7	**3**	BPSV TX09c1.pro
**4**	98.4	97.9	98.4		52.3	52.7	54.9	50.9	52.5	58.7	53.1	50.9	54.5	37.6	53.8	53.6	68.0	52.5	37.2	53.5	28.1	36.8	37.6	27.8	**4**	PCPV VR634.pro
**5**	109.6	110.4	110.9	73.8		99.7	82.2	81.4	90.1	57.0	81.5	81.4	81.5	34.7	83.3	79.2	55.2	81.1	33.7	88.4	28.7	33.7	34.7	28.3	**5**	ORFV B029.pro
**6**	110.9	111.7	112.3	73.0	0.3		82.5	81.1	89.8	57.3	81.8	81.1	81.8	34.7	82.9	79.5	55.6	80.7	33.7	88.4	28.7	33.7	34.7	28.3	**6**	ORFV NZ7.pro
**7**	108.0	104.8	105.4	67.7	20.4	20.0		78.9	84.7	61.0	96.2	80.3	99.3	37.6	86.0	86.3	57.8	84.2	34.3	80.9	30.4	34.0	37.6	30.0	**7**	ORFV NZ2.pro
**8**	98.4	99.1	99.7	77.5	21.4	21.9	24.9		81.2	55.2	79.6	95.7	78.1	37.2	82.5	82.4	53.8	81.8	35.5	84.1	28.1	35.1	37.2	27.8	**8**	ORFV SJ1.pro
**9**	107.7	105.9	106.4	73.3	10.6	11.0	17.2	21.7		59.2	84.0	81.6	84.0	34.5	82.9	84.0	57.6	81.2	33.8	85.8	28.6	33.5	34.5	28.3	**9**	ORFV IA82.pro
**10**	107.1	107.9	108.5	59.2	62.9	62.2	54.5	66.9	58.2		58.8	55.6	60.6	35.7	59.1	59.4	68.3	59.7	34.1	57.3	31.2	33.7	35.7	30.9	**10**	PCPV IT303/05.pro
**11**	109.3	106.1	106.7	72.0	21.3	20.9	4.0	23.9	18.1	59.0		81.7	95.5	35.7	86.0	85.6	56.7	84.6	33.6	80.9	28.9	33.2	35.7	28.5	**11**	ORFV NA1/11.pro
**12**	99.7	100.4	100.9	77.5	21.4	21.9	22.9	4.4	21.2	66.1	21.0		79.6	36.5	86.1	80.3	53.8	86.1	34.3	84.6	28.1	34.0	36.5	27.8	**12**	ORFV Hubei.pro
**13**	109.3	106.1	106.7	68.6	21.3	20.9	0.7	25.9	18.1	55.2	4.7	23.9		37.6	86.0	86.3	57.4	84.2	34.3	80.9	30.4	34.0	37.6	30.0	**13**	ORFV Orf-11.pro
**14**	109.5	112.3	112.3	120.9	134.0	134.0	121.0	122.6	135.2	129.4	129.3	125.9	121.0		35.1	35.4	36.1	36.0	46.5	34.6	30.3	46.2	100.0	29.9	**14**	PVNZ HL953-112.pro
**15**	113.4	110.1	110.7	70.3	19.0	19.4	15.5	20.0	19.4	58.3	15.5	15.5	15.5	132.3		86.1	55.9	93.2	34.1	84.9	28.3	33.7	35.1	27.9	**15**	ORFV SA00.pro
**16**	115.9	111.2	111.7	70.7	24.5	24.0	15.2	20.1	18.0	57.9	16.0	22.9	15.2	130.5	15.5		57.2	87.1	34.1	82.6	27.9	33.7	35.4	27.6	**16**	ORFV NP.pro
**17**	101.4	102.2	102.7	41.7	67.0	66.2	61.3	70.3	61.6	41.1	63.6	70.3	62.0	127.7	65.3	62.5		55.0	35.6	55.3	28.0	35.2	36.1	27.6	**17**	PCPV F00.120R.pro
**18**	111.5	109.5	110.1	73.4	21.9	22.4	17.8	20.9	21.7	57.2	17.3	15.5	17.8	128.2	7.1	14.1	67.3		34.2	84.6	27.7	33.8	36.0	27.3	**18**	ORFV YX.pro
**19**	114.6	125.5	122.2	122.8	139.0	139.0	135.8	130.4	138.3	137.1	139.6	135.8	135.8	89.6	137.1	137.1	129.9	136.4		36.1	27.5	99.6	46.5	27.1	**19**	PVNZ HL953-111.5.pro
**20**	99.1	96.8	97.5	70.9	12.6	12.6	22.1	17.9	15.8	62.3	22.1	17.3	22.1	134.8	16.9	19.8	66.8	17.3	127.4		27.0	36.1	34.6	26.5	**20**	ORFV D1701.pro
**21**	159.3	160.9	159.3	172.5	168.7	168.7	157.6	172.5	169.1	152.3	167.3	172.5	157.6	158.3	171.3	174.0	173.5	175.9	177.2	181.1		27.4	30.1	99.3	**21**	PVNZ HL953-112.5.pro
**22**	116.7	127.7	124.4	124.4	139.0	139.0	137.7	132.2	140.2	139.0	141.5	137.7	137.7	90.7	139.0	139.0	131.6	138.3	0.4	127.4	178.0		46.4	27.0	**22**	PVNZ NZ-112.0
**23**	110.1	112.9	112.9	120.9	134.0	134.0	121.0	122.6	135.2	129.4	129.3	125.9	121.0	0.0	132.3	130.5	127.7	128.2	89.6	134.8	159.0	90.2		29.8	**23**	PVNZ NZ-112.3
**24**	161.6	163.2	161.6	175.2	171.3	171.3	159.9	175.2	171.7	154.5	169.8	175.2	159.9	160.7	174.0	176.7	176.2	178.8	180.1	185.2	0.7	180.9	161.4		**24**	PVNZ NZ-112.6
	**1**	**2**	**3**	**4**	**5**	**6**	**7**	**8**	**9**	**10**	**11**	**12**	**13**	**14**	**15**	**16**	**17**	**18**	**19**	**20**	**21**	**22**	**23**	**24**		
